# *Pitx1* determines characteristic hindlimb morphologies in cartilage micromass culture

**DOI:** 10.1371/journal.pone.0180453

**Published:** 2017-07-26

**Authors:** Natalie C. Butterfield, Chen Qian, Malcolm P. O. Logan

**Affiliations:** 1 Division of Developmental Biology, Medical Research Council – National Institute for Medical Research, London, United Kingdom; 2 Confocal Image Analysis Lab, Medical Research Council – National Institute for Medical Research, London, United Kingdom; Northwestern University, UNITED STATES

## Abstract

The shapes of homologous skeletal elements in the vertebrate forelimb and hindlimb are distinct, with each element exquisitely adapted to their divergent functions. Many of the signals and signalling pathways responsible for patterning the developing limb bud are common to both forelimb and hindlimb. How disparate morphologies are generated from common signalling inputs during limb development remains poorly understood. We show that, similar to what has been shown in the chick, characteristic differences in mouse forelimb and hindlimb cartilage morphology are maintained when chondrogenesis proceeds *in vitro* away from the endogenous limb bud environment. Chondrogenic nodules that form in high-density micromass cultures derived from forelimb and hindlimb buds are consistently different in size and shape. We described analytical tools we have developed to quantify these differences in nodule morphology and demonstrate that characteristic hindlimb nodule morphology is lost in the absence of the hindlimb-restricted limb modifier gene *Pitx1*. Furthermore, we show that ectopic expression of *Pitx1* in the forelimb is sufficient to generate nodule patterns characteristic of the hindlimb. We also demonstrate that hindlimb cells are less adhesive to the tissue culture substrate and, within the limb environment, to the extracellular matrix and to each other. These results reveal autonomously programmed differences in forelimb and hindlimb cartilage precursors of the limb skeleton are controlled, at least in part, by *Pitx1* and suggest this has an important role in generating distinct limb-type morphologies. Our results demonstrate that the micromass culture system is ideally suited to study cues governing morphogenesis of limb skeletal elements in a simple and experimentally tractable *in vitro* system that reflects *in vivo* potential.

## Introduction

Limb morphogenesis involves the spatio-temporal integration of a complex array of signalling factors and morphogen gradients along the three axes of the limb bud [1, 2]. Anterior-posterior patterning is determined by the secretion of the morphogen sonic hedgehog from the zone of polarising activity in the posterior limb bud [3]. This is linked to proximal-distal patterning by a feedback loop between sonic hedgehog and fibroblast growth factors secreted by the distal apical ectodermal ridge [4, 5]. Finally, dorsal-ventral patterning is determined by the restriction of the dorsalising factor Wnt7a in the non-ridge ectoderm (reviewed in [6]). Production of the mature limb requires integration of this complex signalling landscape with finely regulated programs of growth and differentiation. It is currently assumed that key patterning events occur in the same way in the forelimb and the hindlimb. Nevertheless, the final morphology of the forelimb and hindlimb is distinct. How these distinct morphological outputs are derived from common signalling inputs is not understood.

**Fig 1:**
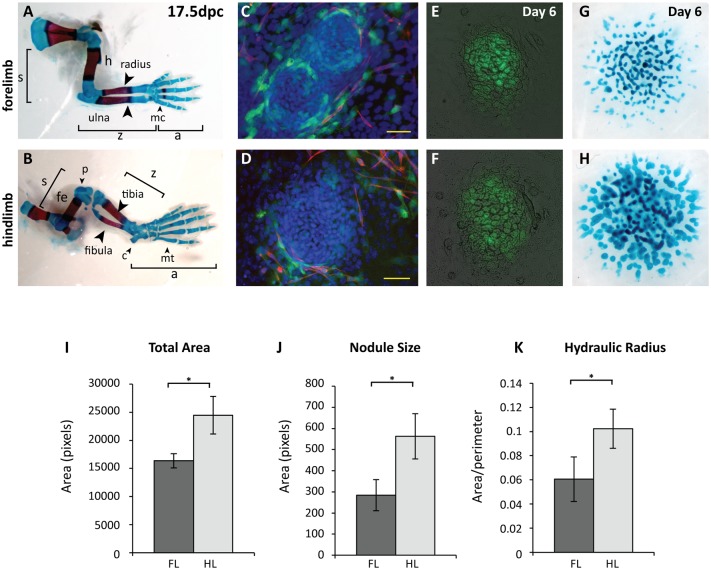
Mouse forelimb and hindlimb micromass cultures display differences in patterning of chondrogenic nodules. **A–B;** 17.5dpc mouse forelimb (**A**) and hindlimb (**B**) showing the three segments (stylopod, s, zeugopod, z and autopod, a) stained with Alcian blue for cartilage and Alizarin red for bone. Forelimb elements include the metacarpals (mc), and humerus (h). Hindlimb elements include the metatarsals (mt), the calcaneus (c), the patella (p) and the femur (f). **C–D;** A single nodule from a day 4 *Scleraxis-GFP* forelimb (**C**) and hindlimb (**D**) micromass showing tenoblasts (Scx-GFP; green), myoblasts (My32, red) and nuclei (DAPI, blue). **E–F;** Day 6 nodules present in forelimb (**E**) and hindlimb (**F**) micromasses display similar nodule makeup. Col2-GFP expression (green) is visible within cartilaginous nodules. **G–H;** Day 6 micromasses established from 11.5dpc mouse forelimb (**G**) and hindlimb (**H**) showing differences in size and shape of cartilage nodules stained by Alcian blue. **I–K;** Quantification of forelimb and hindlimb nodule characteristics across 6 experiments with >6 forelimb and hindlimb micromasses per experiment. Total chondrogenic area (**I**), nodule size (**J**) and hydraulic radius (area/perimeter; **K**) are larger in hindlimb cultures. P< 0.05, Student’s paired *t*-test. C–D; scale = 50μm.

The forelimb and hindlimb are considered serially homologous structures with distinct morphologies that have diverged in parallel with the adaptation of their functions. The mouse hindlimb is overall larger in size and is characterised by elongated metatarsals in the autopod, or footplate (Fig 1B). This elongation is the result of an initial period of rapid growth in the metatarsals compared to the metacarpals [7]. The hindlimb also contains a hindlimb-specific skeletal element, the patella. The non-load bearing fibula is much slimmer than the tibia, while the radius and ulna in the forelimb are approximately equivalent in diameter. The knee joint in the hindlimb and the elbow in the forelimb are also articulated in opposite directions. Finally, the size and shape of the carpal bones of the forelimb and tarsal bones of the hindlimb are distinct, and an example of this is the elongated calcaneus or heel bone in the hindlimb (Fig 1). Such divergence in limb type morphology is thought to arise during limb development from a combination of heterogeneous tissue interactions and the expression of modifying genes. This modifier model postulates that limb-type specific morphologies are a product of the genes whose expression is restricted to either the forelimb or hindlimb (reviewed in [8]). Previous studies have uncovered surprisingly few such genes [9, 10]. Of these, the T-box transcription factors Tbx5 and Tbx4 are absolutely required for the initiation of forelimb and hindlimb budding respectively [11–14]. Despite this, genetic deletion and replacement studies have shown that *Tbx5* and *Tbx4* do not determine limb-type morphologies, although both play key roles later in soft-tissue patterning [14–17]. An alternative model, with both *Tbx4* and *Pitx1* contributing to determining hindlimb morphologies, has also been proposed [18].

The hindlimb-restricted paired-homeobox transcription factor Pitx1 can satisfy the criteria of being a limb-type modifier gene. *Pitx1* deletion results in the loss of some hindlimb skeletal characteristics such as the patella and the differential diameters of tibia and fibula, as well as dysmorphology of the calcaneus and shortening of the metacarpals [7, 19–21]. In addition to sculpting hindlimb skeletal morphology, Pitx1 acts in concert with Tbx4 to ensure correct hindlimb outgrowth [7]. Conversely, ectopic expression of *Pitx1* in the developing forelimb of both chick and mouse transforms the size, shape and position of musculoskeletal tissues of the forelimb into those of the hindlimb [7, 22, 23]. Hence *Pitx1* is both necessary and sufficient to impart hindlimb-type morphologies during limb morphogenesis in both chick and mouse.

The limb skeleton is derived from cartilage anlage formed by aggregation and condensation of mesenchymal precursors into dense pre-chondrogenic condensations expressing type 1 collagen, tenascin-C, hyaluronic acid and fibronectin [24]. These condensed mesenchymal cells then differentiate into chondrocytes, a process requiring Sox9, the SRY-box transcription factor considered to be the master regulator of chondrogenesis [25, 26]. As the cartilage undergoes maturation, chondrocytes proliferate and undergo hypertrophic, and subsequently, terminal differentiation. After the formation of the cartilaginous anlagen, these terminal chondrocytes will eventually undergo apoptosis concomitant with the invasion of osteoblasts and blood vessels to produce mature ossified bone [27, 28]. At the early stages of cartilage formation, the pattern of Sox9-expressing domains is largely indistinguishable between the forelimb and hindlimb, and broadly pre-figures the cartilage elements into which they will differentiate. In the late stages of skeletal development, interaction with the surrounding musculoskeletal system combined with isotropic growth will sculpt the skeletal elements into their final limb-type specific morphologies. Aspects of skeletal shape impacted in this way include bone cross-section and the presence of tuberosities and bone ridges. The presence and morphology of these features is dependent on load imparted by the developing muscles and tendons during late stages of development [29].

The integrated nature of limb bud morphogenesis presents technical challenges when investigating single processes in a complex environment. A need exists for simplified systems to study aspects of limb development, such as cartilage morphogenesis, in isolation. Micromass culture is an experimentally tractable system for studying the cues governing chondrogenesis in a simplified setting [30]. In this system, plating of cells at high density restricts cell spreading, maintains a rounded shape and drives differentiation down the chondrocyte lineage [31, 32]. The system has previously been used to investigate the processes shaping the skeletal elements of the chick wing and leg [33, 34]. Micromass cultures have also been established from forelimbs and hindlimbs of Anolis lizards and cultures can maintain their forelimb or hindlimb-characteristic gene expression patterns for at least 8 days in culture [35]. Wing micromass cultures were shown to contain flat, diffusely-staining cartilage regions, while leg cultures contained larger amounts of cartilage, organised in a nodular pattern [33, 34]. Previous work has also suggested that leg cells are more cohesive than wing cells [33, 36] and implicated the ECM in this phenomenon [33]. It has not been determined if similar phenomena occur in mouse.

We sought to combine the well-characterised micromass system, developed in chick, with the power of mouse genetics to further dissect the generation of limb-type specific morphologies in cartilage development. When mouse forelimb and hindlimb cells are dispersed and cultured *in vitro*, the chondrogenic nodules have a characteristic morphology determined by their limb-type origin. We observed that hindlimb cultures contained characteristically larger and rounder cartilage nodules, and that, overall, more cartilage was produced in hindlimb cultures. We have developed a novel *in silico* analysis pipeline to quantify these differences. Using this system, we demonstrate the dependence of hindlimb-type nodule morphologies in culture on the limb type modifier gene *Pitx1*. We also show that forelimb and hindlimb cells have inherent adhesive differences and that this may be significant in the generation of limb-type morphologies.

## Materials and methods

### Micromass cultures

Chick micromass cultures were performed as previously published [33]. Mouse micromass cultures from forelimbs and hindlimbs were established as previously published [37] with the following modifications. Outbred wild-type Parkes mice were maintained on a 12-hour light/dark cycle, and noon on the day of vaginal plug was designated 0.5 days post coitum (dpc). Autopods were dissected from 11.5–12.0dpc embryos precisely stage-matched by counting the number of tail somites caudal to the hindlimb as described in [38]. Dissociated limb bud cells were diluted to 2 x 10^7^ cells/mL. 10 μL droplets (200 000 cells) were plated in the centre of each well of a multi-well plate (Nunclon Δ-surface, Nunc). After adhering for 1 h at 37°C in 5% CO_2_, droplets were carefully flooded with culture medium and allowed to grow at high density at 37°C in 5% CO_2_, and media changed every 48 h. Day of culture establishment was designated as day 0. Work involving experimental animals (mice) was reviewed and approved by local ethical review panel (ERP) of MRC-NIMR. Work was carried out under appropriate licence from the UK Home Office. Animals were sacrificed using an approved method of cervical dislocation.

#### Immunocytochemistry of micromasses

Micromass cultures prepared as above from Parkes, *Scleraxis-GFP* [39] or *Collagen2-GFP* [40, 41] mice were fixed for 15 min at room temperature with 4% paraformaldehyde (PFA) and rinsed twice with PBS. Cultures were permeabilised for 10 min with 1% Triton X-100/PBS, rinsed twice in PBS and blocked for 30 min in 2% BSA/PBS at room temperature. Primary antibodies were applied for 1 h at room temperature or at 4°C overnight in blocking solution. Primary antibodies were removed by washing 5 times with blocking solution over 30 min, and then cultures were incubated with secondary antibodies conjugated to fluorophores (polyclonal goat α-rabbit-AlexaFluor-488 A-11034, raised against rabbit IgG, Antibody Registry ID AB_2576217 used at 1/200 dilution, and monoclonal goat α-mouse-Alexa-555 A-32727, raised against mouse IgG, Antibody Registry ID AB_2633276 used at 1/200 dilution, both from Molecular Probes) diluted in blocking solution for 1 h at room temperature. Secondary antibodies were removed by washing 5 times over 30 min with blocking solution containing 4’,6-diamidino-2-phenylindole (DAPI, 1/15 000, Roche) to counterstain for nuclei. Cultures were rinsed in distilled water prior to mounting in Prolong Gold (Life Technologies). Confocal Z-stacks of micromass cultures were obtained using a Zeiss confocal microscope with identical parameters across conditions, and images processed using Volocity software (Perkin Elmer) and Adobe Photoshop CS5.1. Primary antibodies used: α-fibronectin [monoclonal mouse, ab131065 (Fn3E2), raised against full length human fibronectin, Antibody Registry ID AB_11156091 used at 1/200 dilution, Abcam], α-tenascin-C; [monoclonal rabbit, ab108930 (EPR4219), raised against a synthetic peptide from Human Tenascin C, Antibody Registry ID AB_10865908 used at 1/100 dilution, Abcam], α-fast skeletal muscle [monoclonal mouse M4276 (My-32), raised against rabbit muscle myosin, Antibody Registry ID AB_477190 used at 1/500, Sigma], α-alpha tubulin [monoclonal mouse T9026 (DMIA), raised against chicken brain tubulin, Antibody Registry ID AB_477593 used at 1/1000, Sigma] and α-GFP (polyclonal rabbit A-21311 conjugated to AlexaFluor-488, raised against *Aequorea victoria* Green Fluorescent Protein, Antibody Registry ID AB_221477, used at 1/200 dilution, Molecular Probes).

#### Staining of micromass cultures for cartilage

Fixed cultures were incubated in two changes of 0.1N HCL over 10 min, followed by incubation with 0.1% Alcian blue GX (Fluka)/0.1N HCL overnight at room temperature. Under these conditions, Alcian blue stains sulphated proteoglycans expressed by mature chondrocytes. Stain was removed by 2 x 5min washes in 70% ethanol and photographed in 70% ethanol using a Leica DFC320 camera with 1x objective and Leica Firecam software, or an Epson scanner (for quantification; see below). Cultures were dried for long-term storage at room temperature.

#### *Pitx1*^*-/-*^ and *Prx1-Pitx1* transgenic micromasses

*Pitx1*^*+/-*^ males [20], maintained on an SV129 background were crossed with *Pitx1*^*+/-*^ females on an MF1 background. Micromasses were established from limb buds obtained from pooled control and *Pitx1*^*-/-*^ stage-matched littermates at 11.5dpc as for wild-type micromasses and fixed and stained with Alcian blue at day 7 to visualise mature cartilage. Transgenic mice ectopically expressing the *Pitx1* gene in the forelimb field (*Prx1-Pitx1*^*Tg/WT*^) were produced by inserting the mouse *Pitx1* coding region downstream of the *Prx1* enhancer element described in [23]. *Prx1-Pitx1*^*Tg/WT*^ mice were maintained on a Parkes background. *Prx1-Pitx1*^*Tg/WT*^ males were crossed with Parkes females and embryos harvested at 11.5dpc as for wild-type micromasses. As transgenic and control littermates at this stage cannot be distinguished with reasonable accuracy, individual limb bud pairs were cultured separately. The micromass protocol was modified to suit the lower amount of starting material as below. Limb pairs from individual mice were treated as biological replicates. Limb pairs were incubated in 10 mg/mL dispase II (Roche), 10 mM Hepes pH 7.4 for 30 min at 37°C, and ectoderm manually removed with fine forceps. Limb samples were diluted and triturated to generate a single cell suspension before cell counting and resuspension to a concentration of 2 x 10^7^ cells/mL and plating as above.

#### Quantification of micromass characteristics

Micromasses cultured in multi-well tissue culture plates were imaged simultaneously by scanning whole plates at very high resolution using an Epson Perfection V750 Pro document scanner (2 400 dots per inch). The images were converted to HSB stack mode using ImageJ (NIH), and the black and white saturation layer was imported into Labview 2009 (National Instruments) for quantification of culture parameters. All well positions were first detected, and regions of interest (ROI) around each culture were defined. Alcian blue-positive regions in each culture were selected automatically a using local threshold. This threshold was identical across all biological and technical replicates within each experiment. Detection of well positions and thresholding of cultures were performed using Niblack’s local threshold algorithm (NiBlack, 1986). This algorithm is particularly suited to thresholding against a variable background, caused by shadowing from tissue culture well sides and any uneven illumination across the tissue culture plate. Local threshold value (T) is based on the equation:

T(x,y)=m(x,y)+k⋅s(x,y),

where *m* is the average and *s* is the standard deviation of the grey values in a sliding window (*x*, *y*). Size of the sliding window was optimised to represent local details while excluding noise. The deviation factor *k* determines how close each pixel must be to the local mean to be selected as part of a particle. The size of the sliding window and the deviation factor were kept constant for each well, and also between experiments. Once areas for analysis had been thresholded, boundaries were smoothed using 10 iterations of a dilation function (for well detection) and an auto-median function (for cartilage nodule detection) to generate nodule boundaries and remove background noise. Detected cartilage nodules were filtered for size based on previous assessment of nodule dimensions, and particles above 40 pixels in size were counted as nodules. Parameters collected (in number of pixels) for each culture were: total chondrogenic area, nodule size and hydraulic radius (area of nodule/perimeter; weighted for particle size).

Each experiment was treated as a biological repeat and each micromass culture within the experiment was treated as a technical repeat. Mean and standard deviation for each condition was determined in Microsoft Excel. Statistical significance was determined using unpaired two-tailed Student’s *t*-tests assuming unequal variance, P***<0.001, P**<0.01 and P*<0.05. For *Prx1-Pitx1*^*Tg/WT*^ micromasses, individual limb pairs were plated separately and biological replicates (complete with 1–4 technical repeats) were obtained within one experiment, and that experiment was repeated 4 times. To represent biological variation, pooled standard deviation was calculated from biological repeats of control and transgenic micromass samples. To compare pooled means, Student’s *t*-test (two-tailed, unequal variance) was used to detect statistically significant differences between control and transgenic samples for each experiment. For wild-type forelimb and hindlimb comparisons, a total of 6 biological replicates were obtained, with each replicate consisting of >6 technical replicates. For *Pitx1*^*-/-*^ forelimb and hindlimb comparisons, biological triplicates were obtained, each consisting of >4 technical replicates. For *Pitx1*^*Tg/WT*^ forelimb and hindlimb comparisons, biological quadruplicates were obtained, each consisting of 2–4 technical replicates.

#### Micromass-based substrate adhesion assay

Micromasses from forelimbs and hindlimbs of wild-type 11.5dpc Parkes embryos were established as above. Immediately after cultures were flooded with medium (after drops had adhered to culture dishes for 1 hour), cultures were subjected to an identical adhesion challenge (5 shakes and 1 gentle trituration). Control cultures were fixed (see below) without the adhesion challenge to provide total cell numbers. Cultures were incubated with Hanks Balanced Salt Solution (HBBS; Sigma) containing 0.6% methylene blue (BDH Chemicals)/1.25% glutaraldehyde (Sigma) for 1 h at 37°C and 5% CO_2_. Cultures were rinsed thoroughly in dH_2_O and dried prior to elution of bound methylene blue stain by incubation in 50% ethanol/1% acetic acid/49% PBS for 2 h rocking at room temperature. Dissolved dye samples were read at OD_650_ and cell number was calculated from a standard curve [42]. To buffer for plating variation between samples, cell numbers remaining on plates after the adhesion challenge were represented as a percentage of the number of cells in micromasses fixed and stained without the adhesion challenge. The experiment was repeated three times with technical replicates of 4–6 forelimb and hindlimb micromasses per experiment.

### *In vivo* dispase dissociation assays

Wild-type 11.5dpc Parkes forelimb and hindlimb buds were harvested in Puck’s saline A (5.4 mM KCl/140 mM NaCl/4.2 mM NaHCO3/6.1 mM glucose [43]). Quadruplicate pools of >4 forelimb and hindlimb buds were partially dispersed by passing twice through a 22G needle, followed by a short digestion for 10 min at 37°C in 1 U/μL dispase II/10% HI-FBS/Puck’s saline A shaking at 300 rpm. Limb buds were triturated once with a 1 000 μL pipette, and the number of single cells released from each limb bud pool was determined by haemocytometer. To obtain total cell numbers present in each limb bud, partially-digested limb buds were then pelleted at 700 g (1 200 rpm) for 3 min and resuspended in 0.05% trypsin-EDTA (Gibco) for complete digestion over 20 min at 37°C. Cell counts at both stages were blinded. The partial dissociation of forelimb and hindlimb samples (represented by single cell numbers released by the short dispase digestion) was represented as a percentage of the final cell number (represented by the cell numbers after complete digestion of the limbs by trypsin) to buffer for variations in staging and starting material. Mean and standard deviation were calculated, and statistical significance was determined using unpaired Student’s *t*-tests (two-tailed, unequal variance; p*<0.05, p**<0.01, p***<0.001).

### Skeletal preparations

Mouse forelimb and hindlimb skeletons at 17.5dpc were stained for cartilage and bone with Alcian blue and Alizarin red respectively as previously published [44].

### Western blots

Whole limbs were lysed in radio-immunoprecipitation (RIPA) buffer (50mM Tris pH 7.5/150 mM NaCl/0.1% SDS/0.5% sodium deoxycholate/1% NP-40) with Complete mini protease inhibitor tablets (Roche) and extracted on ice for 15 min. Samples were centrifuged at 13 000 rpm for 15 min and the soluble supernatant was diluted in 5x Laemmlie sample buffer (60 mM Tris pH 6.8/% SDS/10% glycerol/0.1% bromophenol blue) with 5% β-mercaptoethanol and boiled for 5 min at 95°C. Protein concentration was determined by BCA assay (Pierce). Total protein (5–10 μg) was loaded onto 7% Nupage Novex tris-acetate gels with tris-acetate running buffer (Life Technologies) and transferred onto nitrocellulose membrane using Nupage transfer buffer containing 20% methanol and antioxidant at 15 V overnight at 4°C (Life Technologies). Blocking buffer [5% skim milk powder (Marvel)/PBS/0.1% Tween-20] was used to block the membrane for 1 h at room temp and dilute primary and secondary antibodies. Antibodies were rinsed by 5 washes of PBS/0.1% Tween-20 over 30 min at room temperature. Primary antibodies α-tenascin-C [monoclonal rabbit, ab108930 (EPR4219), raised against a synthetic peptide from Human Tenascin C, Antibody Registry ID AB_10865908 used at 1/100 dilution, Abcam] and α-alpha tubulin [monoclonal mouse T9026 (DMIA), raised against chicken brain tubulin, Antibody Registry ID AB_477593 used at 1/1000, Sigma] were applied overnight at 4°C or for 2 h at room temperature, and horseradish peroxidase-conjugated secondary antibodies (polyclonal rabbit α-mouse-HRP, P0161, raised against immunoglobulins isolated from mouse serum, used at 1/1 000 dilution, DAKO), and donkey α-rabbit-HRP, NA934, Antibody Registry ID AB_772206, raised against rabbit IgG, used at 1/5 000 dilution, GE Healthcare) were applied for 1 h at room temperature. HRP was detected using ECL western blotting reagents (GE Healthcare) and Kodak Hyperfilm.

## Results

### Forelimb and hindlimb micromass cultures display characteristic nodule morphologies

Micromasses cultures are heterogeneous, and while the culture conditions encourage the growth of chondrogenic nodules, precursors for tendons, muscle and muscle connective tissue are also present. We examined whether the components of forelimb and hindlimb cultures are similar. After three days of culture, chondrogenic nodules are visible as condensations of nuclei, and tendon progenitors, marked by *Scx-GFP*-positive cells (green) are present within and around nodules (Fig 1C and 1D), interestingly particularly around the nodule periphery. Myoblasts (red; Fig 1C and 1D) do not contribute to chondrogenic nodules and are visible in non-condensed internodular regions. Myoblasts do not survive well in the micromass environment, and by day 7 very few myoblasts are present in micromasses (data not shown). Chondrogenic nodules can be identified by expression of GFP driven by the collagen II promoter in differentiated chondrocytes (Fig 1E and 1F). No differences in the overall morphology of *Col2-GFP*-postive cells were observed in forelimb and hindlimb nodules, suggesting that the process of chondrogenesis occurs similarly in forelimb and hindlimb cultures. We next examined nodule morphology by staining with Alcian blue for the sulphated proteoglycans secreted by mature chondrocytes (Fig 1G and 1H). When forelimb and hindlimb micromasses are cultured separately, a striking difference in chondrogenic nodule morphology is evident. Hindlimb cultures are larger overall and individual cartilage nodules also appear larger and rounder than those in forelimb cultures (Fig 1G and 1H). Overall, these data indicate that while the cellular composition of forelimb and hindlimb micromasses is similar, the shape of chondrogenic nodules formed by forelimb or hindlimb cultures differ significantly.

We quantified the characteristic morphologies of forelimb and hindlimb micromasses by developing a pipeline to assess parameters across multiple replicates of forelimb and hindlimb cultures in parallel. This assay is based on quantifying the patterns formed by chondrogenic material stained with Alcian blue. Statistically significant differences were observed in the total chondrogenic area, the size of the nodules, and the hydraulic radius of the nodules present in each culture (Fig 1I and 1K, p*<0.05). Hydraulic radius is calculated by dividing the area of each nodule by its perimeter. A large hydraulic radius indicates a smooth, rounded nodule, and a smaller value indicates a more irregular nodule. Hindlimb cultures were observed to have an overall larger total chondrogenic area, and individual nodules present in the culture were larger than forelimb nodules (Fig 1I and 1J). Hindlimb nodules also had a larger hydraulic radius, indicating rounder nodules (Fig 1K). Overall, the patterns formed by chondrogenic nodules of the forelimb and hindlimb *in vitro* display consistent and quantifiably different patterns.

**Fig 2:**
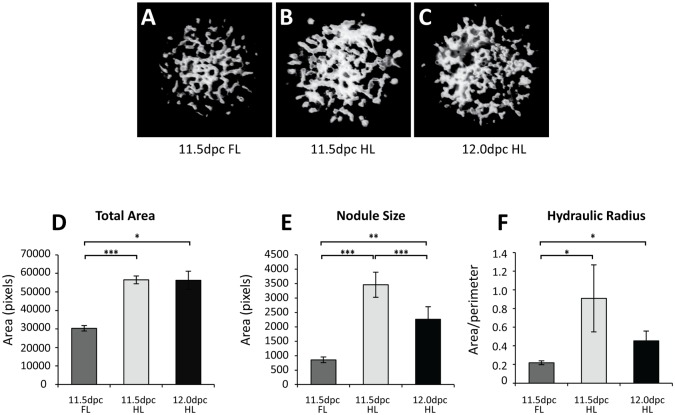
Forelimb and hindlimb-type cartilage morphologies are not the result of heterochrony. **A–C;** Micromasses were established from forelimbs (**A**) and hindlimbs (**B, C**) of embryos 0.5dpc apart (11.5dpc; **A–B** and 12.0dpc; **C**). Total chondrogenic area, nodule size and hydraulic radius of 11.5dpc forelimb and hindlimb cultures were significantly different (**D–E**). Nodules in 12.0dpc hindlimb cultures had significantly larger total area (**D**), nodule size (**E**) and hydraulic radius (**F**) than 11.5dpc forelimb nodules. 11.5dpc HL and 12.0dpc HL cultures displayed similar values for total area (**D**) and hydraulic radius of nodules (**F**). Nodule size was significantly larger in 12.0dpc HL cultures than in 11.5dpc FL cultures, although smaller than in 11.5dpc HL cultures. *p<0.05, **p<0.01, Student’s *t*-test.

To establish that any difference observed in limb chondrogenic patterns is not the result of the approximately 12-hour delay in the budding of the hindlimb compared to the forelimb [45], we compared the nodule morphology in cultures established from limbs of equivalent developmental age to determine if similar patterns were observed in forelimbs and hindlimbs regardless of stage. Micromasses were established from embryos staged 12 hours apart (11.5dpc forelimb and hindlimb and 12.0dpc hindlimbs, which are similar in age to 11.5dpc forelimbs). As found previously, nodules present in cultures established from 11.5dpc hindlimbs are larger, rounder (larger hydraulic radius) and cover a greater total area than 11.5dpc forelimb culture nodules (Fig 2A, 2B and 2D–2F). In contrast, the total area and hydraulic radius of nodules in 11.5dpc hindlimb and 12.0dpc hindlimb cultures are not significantly different. While nodules in 12.0dpc hindlimb cultures were in general smaller than those in 11.5dpc hindlimb cultures, importantly, they were significantly larger, rounder and covered a greater total area than nodules in 11.5dpc forelimb cultures. We conclude that the forelimb/hindlimb differences in cartilage nodule morphology detectable *in vitro* are not a product of heterochrony in limb budding.

### Limb-type nodule morphologies are dependent on *Pitx1*

**Fig 3:**
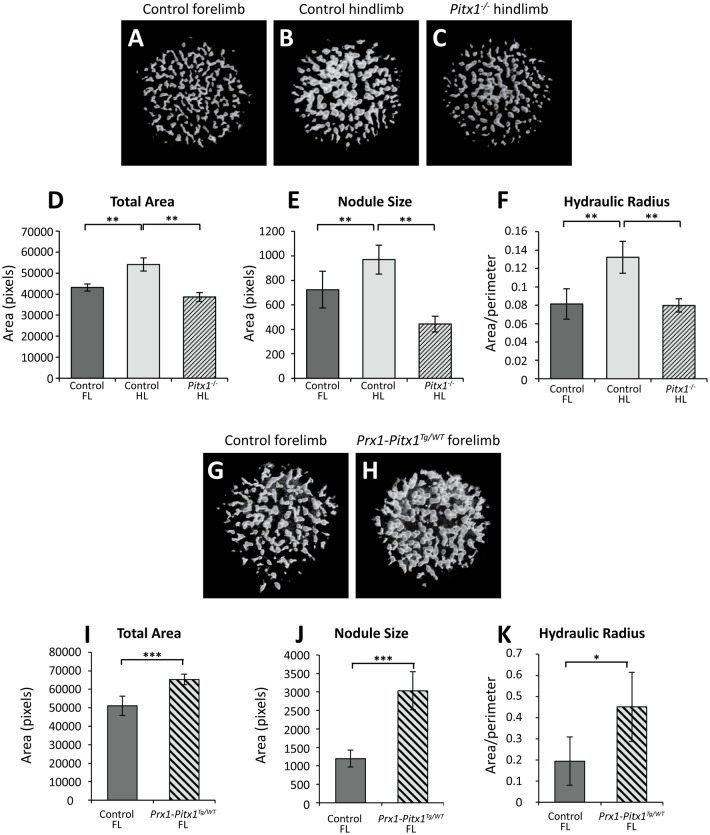
*Pitx1* determines nodule morphology *in vitro*. **A–C;** Representative black and white images of 11.5dpc control forelimb (**A**) and hindlimb (**B**) and *Pitx1*^*-/-*^ hindlimb (**C**) micromasses stained for Alcian blue. **D–F;** Quantification of samples represented by A–C. Chondrogenic nodules in micromasses established from control hindlimbs (**B**) have larger total area, are larger in size (both measured by number of pixels) and have a larger hydraulic radius (**D–F**) than control forelimbs (**A**). In contrast, nodules in micromasses established from *Pitx1*^*-/-*^ hindlimbs (**C**) are significantly smaller, cover less total area, and have a smaller hydraulic radius than control hindlimb cultures (**B**, **D–F**) Control FL and HL; n = 6, *Pitx1*^*-/-*^ HL; n = 4. **G–H;** Micromasses generated from *Prx1-Pitx1*^*Tg/WT*^ forelimbs (**H**) which ectopically express *Pitx1* contain nodules which are larger and have a higher hydraulic radius, and cover a larger total area than control forelimb cultures (**G**, **I–K**). *p<0.05, **p<0.01, ***p<0.001, Student’s *t*-test.

To investigate possible candidates for the production of distinct patterns in forelimb and hindlimb micromass cultures, we examined the influence of a known limb-type modifier gene, *Pitx1*. We quantified micromass nodule morphology in 11.5dpc *Pitx1*^*-/-*^ and control forelimbs and hindlimbs. As observed in wild-type cultures, control hindlimb cultures contained a significantly greater total chondrogenic area, with larger and rounder nodules than forelimb cultures (Fig 3A, 3B and 3D–3F). In striking contrast, nodules in *Pitx1*^*-/-*^ hindlimb micromass cultures are significantly smaller, less round (smaller hydraulic radius) and cover a lesser total area than those present in control hindlimb cultures (Fig 3B, 3C and 3D–3F). These data demonstrate that in the absence of *Pitx1*, the significant difference between chondrogenic nodule size and shape detectable in control forelimb and hindlimb cultures is greatly reduced and that *Pitx1*^*-/-*^ hindlimb micromass cultures morphology is most similar to forelimb micromass cultures.

We next investigated whether the ectopic expression of *Pitx1* in the non-*Pitx1*-expressing forelimb is sufficient to induce changes in forelimb micromass cultures to make them hindlimb-like. Micromass nodule morphology was quantified in cultures established from control and *Prx1-Pitx1*^*Tg/WT*^ forelimbs. Nodules in *Prx1-Pitx1*^*Tg/WT*^ forelimb cultures were significantly larger and rounder (larger hydraulic radius) and overall covered a greater total chondrogenic area compared to control forelimbs (Fig 3G, 3H and 3I–3K). Taken together, these data indicate that presence and absence of *Pitx1* is directly correlated with the generation of limb-type micromass nodule morphologies.

### Forelimb and hindlimb cells display inherently different properties

**Fig 4:**
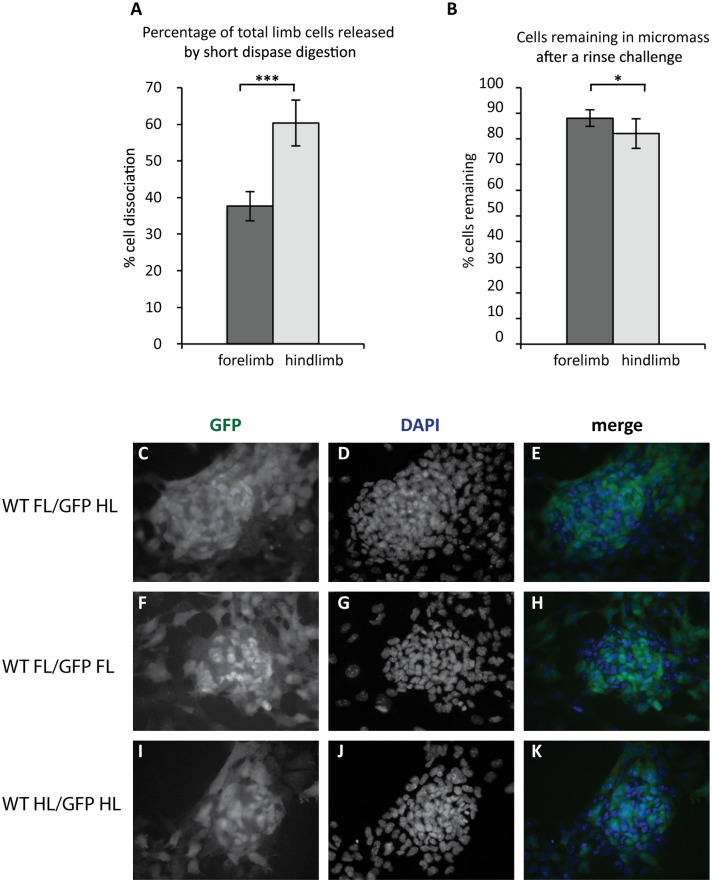
Hindlimb cells are more adhesive *in vivo* and *in vitro* than forelimb cells. **A**) Enzymatic dissociation of 11.5dpc limb buds with dispase releases more hindlimb cells than forelimb cells (p = 0.0021, n = 6). **B**) When micromass cultures are challenged after 1 hour of adhesion to the cell culture substrate, fewer hindlimb cells are left in the culture than forelimb cells (p = 0.011, n = 4 pools of >4 limbs each, normalised for limb number). *p<0.05, **p<0.01, ***p<0.001, Student’s *t*-test. **C–K;** Cell mixing experiments, 1:1 mix of *Prx1eGFP* hindlimb cells/wild type forelimb cells (**C–E**), *Prx1-eGFP* forelimb/wild type forelimb (**F–H**) and *Prx1-eGFP* hindlimb/wild type hindlimb (**I–K**) cells were stained for GFP protein (green) and nuclei (blue, DAPI).

A critical factor in the process of chondrogenesis is adhesion of cartilage precursors to each other and to the extracellular matrix. We next assessed whether forelimb and hindlimb buds display different adhesive properties *in vivo* by quantifying the percentages of 11.5dpc forelimb and hindlimb buds dissociated from the limb bud by enzymatic digestion. Significantly more cells were released from hindlimbs (60.3%) than from forelimbs (37.6%; Fig 4A, p = 0.0021) under the same dissociation conditions. This suggests that hindlimb cells are less cohesive than forelimb cells at this stage.

We next investigated whether hindlimb cells are more cohesive to each other and to the culture substrate *in vitro* by examining the ability of forelimb and hindlimb cells to attach under the conditions of the micromass protocol. Forelimb and hindlimb micromasses were subjected to a standardised adhesion challenge 1 hour after plating, and the numbers of cells remaining on the dish were quantified. Numbers of remaining cells were represented as a percentage of the number of cells in micromasses not subjected to an adhesion challenge (Fig 4B). Fewer hindlimb cells (82.1% of the culture) were left on the dish after the adhesion challenge than forelimb cells (88.1%; p = 0.012). This suggests that during this early time window of culture, cells in hindlimb micromasses are less adhesive to each other and to the culture substrate than those in forelimb micromasses.

To further explore possible differences in adhesive properties of forelimb and hindlimb cells we assessed whether forelimb and hindlimb cells undergo cell mixing when brought together outside the limb environment. Primary monolayer cultures were established from 1:1 mixtures of *Prx1;eGFP*^*Tg/WT*^ hindlimb/wild-type forelimb cells. No segregation of eGFP-expressing and wild-type cells was observed over a culture period of 5 days (Fig 4C–4K). Next, micromasses were established from either forelimb or hindlimb cells from chick embryos expressing ubiquitous cytoplasmic GFP [46] in a 1:1 mix with wild-type forelimb or hindlimb cells. No segregation of wing and leg cells from the same proximal-distal level was observed after 3, 5 and 8 days of culture. This is in agreement with previous studies demonstrating that mixed cultures containing proximal and distal chick cells show segregation, but heterotypic mixes of forelimb and hindlimb cells from the same proximal-distal level do not [47, 48]. Therefore although our data indicate that hindlimb cells seem inherently less cohesive in the limb and *in vitro*, forelimb and hindlimb cells do not segregate in either chick or mouse cultures.

Given the observed differences in adhesive properties between forelimb and hindlimb cells, we investigated whether modulation of the ECM might be implicated in the generation of limb-type morphologies *in vitro*. We observed no difference in level or expression pattern of fibronectin by immunocytochemistry in day 3 micromass cultures (S1A–S1F Fig), nor of Tenascin-C [49–53] in 11.5dpc mouse forelimbs and hindlimbs by western blot (S1G Fig). Differences in localisation and/or protein expression levels of fibronectin and tenascin-C are therefore not responsible for the differences in cell-cell adhesion observed.

## Discussion

We demonstrate that when removed from the *in vivo* limb bud environment and cultured *in vitro* at high density, forelimb and hindlimb cells produce distinct and quantifiable differences in cartilage nodule patterns. Chondrogenic nodules forming in hindlimb cultures are larger and rounder and cover a greater total area than those present in forelimb micromass cultures. We have shown that this is due to the actions of *Pitx1*, as these hindlimb nodule characteristics are lost in hindlimb cultures generated from limbs lacking *Pitx1*. Furthermore, we show that forelimb micromass cultures established from forelimbs ectopically expressing *Pitx1* in the forelimb assume hindlimb-type nodule characteristics. The system we have established represents an ideal setting for the investigation of modulators of chondrogenic pattern downstream of *Pitx1*, which we propose may be involved in cellular adhesion during chondrogenesis.

### Forelimb and hindlimb micromass cultures display characteristic limb-type morphologies

We show that micromass cultures established from the forelimbs and hindlimbs of mid-gestation mice display consistent and characteristic differences in cartilage nodule morphology. We have developed a quantification pipeline for parallel analysis of chondrogenesis in micromass culture, and are able to quantify these differences. Our system shows that nodules in micromass cultures established from 11.5dpc mouse hindlimbs are larger and rounder than those in forelimb cultures. Total area covered by chondrogenic material is also greater in hindlimb cultures than in forelimb cultures. Our results indicate that when micromasses are established from limb buds that are 0.5dpc apart in embryonic age (11.5dpc vs 11.0dpc), the differences in nodule characteristics are still maintained. Regardless of any contribution to nodule size by heterochrony, a clear difference is observed between control and genetically modified homotypic limbs. Hindlimbs from stage-matched control and *Pitx1*^*-/-*^ littermates are directly comparable, and we observe consistent alterations in hindlimb nodule morphology in the absence of *Pitx1*. Similarly, we observe consistent and quantifiable differences in forelimb nodule morphology in the presence of ectopic *Pitx1*.

We describe a systematic quantification pipeline to evaluate morphological parameters of micromass cultures in parallel. The assay is based on the detection of mature cartilage in micromass cultures by Alcian blue staining of mature chondrocytes. In some cases we observed a small amount of background staining in cultures but the thresholding process was successful in eliminating this. For this reason, determination of the lower detection threshold, above which Alcian blue-positive regions were counted, was adjusted manually for each experiment. This ensured the thresholded region most closely represented genuine staining as determined by eye. Importantly, threshold conditions across each technical replicate and experimental condition within the experiment were identical. Parameter outputs from the quantification program can therefore confidently be compared within each experiment. Consistency within each experiment is ensured by a) precisely stage-matching embryos using tail somite number b) keeping cell numbers in each micromass identical c) comparing groups within experiments only (wild-type and mutant, forelimb and hindlimb). However, subtle differences in results between experiments can be caused by variation in ages of embryos and batches of reagents such as culture serum and enzymes. For these reasons, and similar to other *in vitro/ex vivo* experiments, actual parameter values are not always identical (compare y-axes in Figs 1I–1K to 2D and 2E). Despite this, in pooled data from 6 independent experiments, the differences in nodule morphology are maintained with statistical significance (Fig 1I–1K).

### Hindlimb-specific micromass morphologies are dependent on *Pitx1*

We demonstrate that micromasses established from *Pitx1* mutant hindlimbs have smaller nodules that are more irregular and contain overall less total chondrogenic area than control hindlimb cultures. These differences in module pattern reflect closely the *in vivo* situation in which *Pitx1* is influential in sculpting the shape and size of the skeletal elements of the hindlimb. Lack of *Pitx1* in mice results in the loss of several characteristics of the hindlimb skeleton [19–21]. This includes loss of hindlimb-specific skeletal elements such as the patella, and an abnormally shaped calcaneus. In the hindlimb, the diameter of the fibula is approximately half that of the tibia while the diameter of the homologous zeugopodal bones of the forelimb (the radius and ulna) are approximately equivalent. Absence of *Pitx1* in the hindlimb results in the loss of this difference in size of the tibia and fibula, with both bones now appearing more or less equal in diameter [7]. Skeletal patterning defects in *Pitx1*^*-/-*^ hindlimbs are accompanied by defects in skeletal muscle and tendon patterning, indicating that Pitx1 is also required to pattern these tissues [7].

Analysis of the *Pitx1*^*-/-*^ phenotype is complicated by the contribution of the hindlimb-restricted T-box transcription factor Tbx4. *Pitx1* is required for *Tbx4* expression, and *Pitx1*^*-/-*^ hindlimbs express hypomorphic levels of *Tbx4* [7, 19, 20]. Transgenic delivery of *Tbx4* in the background of the *Pitx1*^*-/-*^ mutant can rescue the hindlimb outgrowth defect but not hindlimb morphologies, including the articulation of the knee joint, the presence of the patella and the correct morphology of the calcaneus [7]. We have not determined whether the *Pitx1*^*-/-*^ mutant hindlimb micromass cultures express hypomorphic levels of *Tbx4* and if so, what effect this may have on growth of the cultures but we predict that it would have no effect on nodule morphology.

The cartilage nodule morphology observed in *Pitx1*^*-/-*^ hindlimb micromass cultures is similar to that of forelimb micromass cultures (Fig 2). Examination of the *Pitx1*^*-/-*^ hindlimb phenotype *in vivo* demonstrates that the absence of *Pitx1* does not generate forelimb-type morphologies, as would be predicted if forelimb morphology was the default and hindlimb morphology the derived state [18]. Rather, the *Pitx1*^*-/-*^ phenotype represents a loss of hindlimb-type morphologies without the acquisition of forelimb-type morphologies [7]. Therefore the forelimb and hindlimb morphologies each represent derived states rather than the hindlimb deriving from a default forelimb state.

Forelimbs in which *Pitx1* is ectopically expressed display forelimb-to-hindlimb transformations of hard and soft tissues of the forelimb. These include transformation of the elbow joint to resemble a knee joint, and of carpal bones to tarsal bones and alterations in muscle and tendon patterning [7, 23]. Consistent with this, micromass cultures established from *Prx1-Pitx1*^*Tg/WT*^ forelimbs exhibit chondrogenic nodules that are larger and rounder, and overall cover a greater area than wild-type forelimb nodules, a pattern similar to hindlimb micromass nodules. From these overall data, we conclude that Pitx1 is a key cell autonomous driver of hindlimb morphology and can affect micromass nodule morphology *in vitro* away from the limb signalling environment.

### Contribution of other cell types to limb-type morphologies

Micromass cultures contain a heterogeneous mix of tissue precursors, including muscle precursors that migrate into the limb autopod from their origin in the dermomyotome [54]. Muscle cells have previously been implicated in influencing nodule morphology in chick micromasses [55]. We observe a significant number of myosin-positive cells present in micromasses at day 1–3 in both the forelimb and hindlimb during the time period in which the cartilage nodule pattern is being laid down. However, very few myosin-positive cells were present in day 7 cultures, indicating that these cells not survive the micromass protocol (data not shown). Micromass cultures also contain tendon progenitors or tenocytes, and it is likely that a significant proportion of internodular cells are of this lineage. Micromasses established from forelimbs and hindlimbs of embryos expressing GFP under the Scleraxis promoter [39] display GFP-positive cells within and around chondrogenic nodules (Fig 1). The thin layers of Scx-GFP-positive cells around the developing nodules most likely represent nascent tendon structures, although Scx-GFP cells also appear to be capable of contributing to the cartilage nodule itself (Fig 1), consistent with recent studies of Scx-positive tenocytes contributing to bone ridges [56]. It is known that the percentage of non-chondrogenic cells (specifically tenocytes) present in a micromass culture has an impact on nodule size and total chondrogenic area [57]. Whether the proportion of tenocytes is altered in *Pitx1*^*-/-*^ and *Prx1-Pitx1*^*Tg/WT*^ limbs is as yet unknown, and the micromass system is ideally suited to investigating this.

### Hindlimb cells are less adhesive than forelimb cells at 11.5dpc

Cell-cell and cell-matrix adhesion is a key requirement and modulator of pattern generation. Hindlimbs appear intrinsically less cohesive than forelimbs at 11.5dpc, from a dissociation assay using the protease dispase. Dispase is a neutral metalloprotease which has been shown to cleave fibronectin and collagen IV [58]. We found that dispase treatment releases more cells from the 11.5dpc hindlimb than the forelimb. It has been previously shown that fibronectin protein and RNA levels are elevated in chick hindlimbs and 2.5-day hindlimb-derived micromasses compared to forelimbs [33, 34]. However, in the mouse, we observe similar expression of fibronectin protein in day 3 mouse forelimb and hindlimb micromasses. Given the influence of ECM molecules during chondrogenesis, other components of the ECM remain strong candidates for the generation of limb-type specific morphologies during limb development.

In summary, we have established a quantitative system for the study of inherent differences in forelimb and hindlimb chondrogenic precursors. We have demonstrated that the differences in the morphology of skeletal elements that form from chondrogenic precursors in forelimb and hindlimb buds translates to limb type-specific differences in nodule morphologies when these cells are cultured *in vitro*. The micromass system is a tractable system amenable to experimental manipulations, and highly suited to the investigation of events at the cellular level. In particular, the system is suited to the investigation of candidate ECM molecules in the generation of limb-type morphologies and chondrogenesis more broadly *in vitro*.

## Supporting information

S1 Fig Expression patterns and levels of candidate modulators of adhesion are not altered in forelimbs and hindlimbs. **A–F;** Fibronectin expression (red, **A**, **D**) is expressed similarly in forelimb (**A–C**) and hindlimb (**D–F**) day 3 micromasses. Cultures are counterstained with DAPI for nuclei (blue, **B, E**), scale = 70μM. **G;** Tenascin—C protein levels are equivalent in mouse 11.5dpc forelimb and hindlimb buds by western blot. Equal protein loading is shown by reprobing for α–tubulin.
(TIF)
