# Supplementary figures and images for: Pitx1 determines characteristic hindlimb morphologies in cartilage micromass culture
Source: PLoS One. 2017 Jul 26;12(7):e0180453. doi: 10.1371/journal.pone.0180453 (PMC5528256; doi:10.1371/journal.pone.0180453)

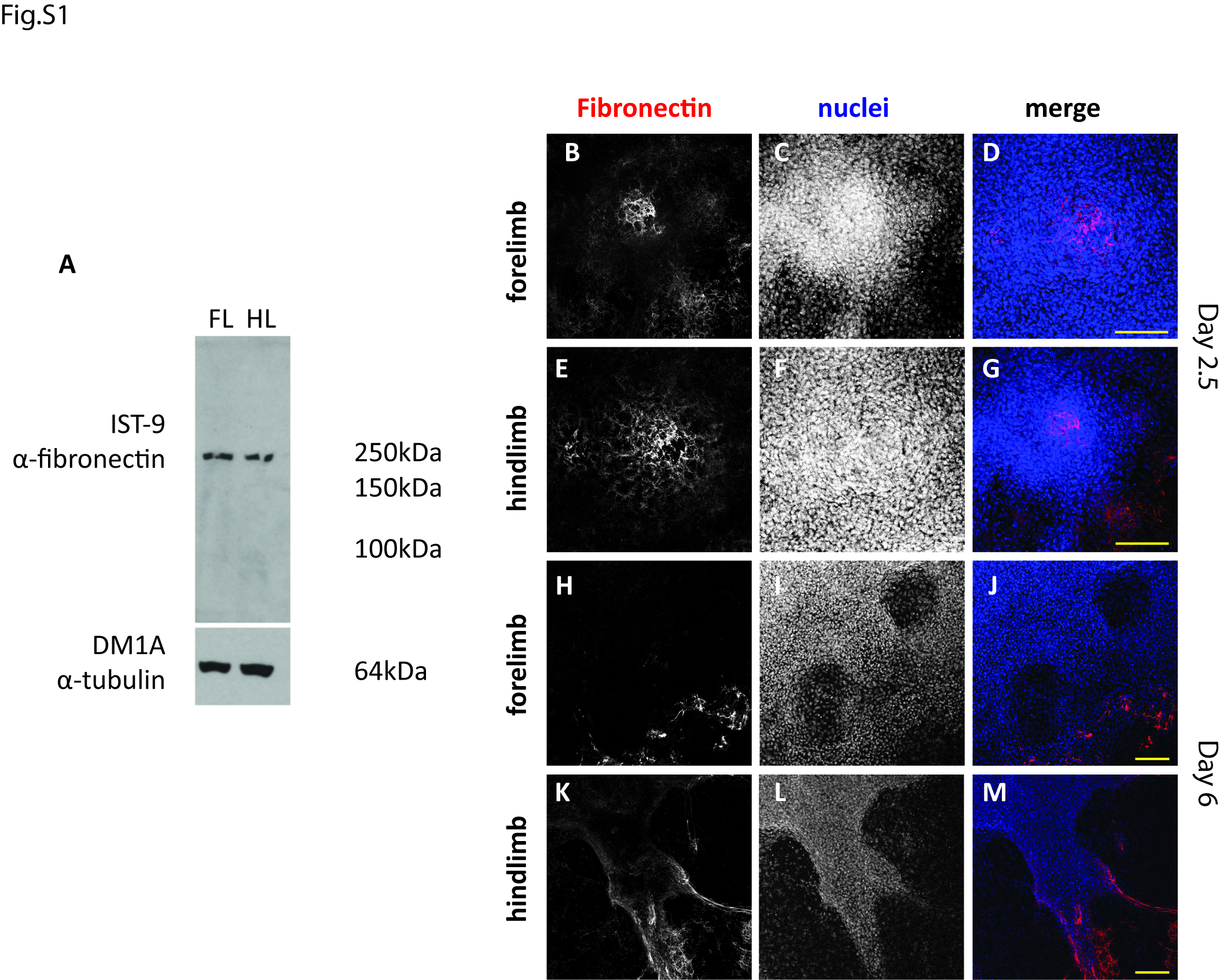

Supplement: S1 Fig — A–F; Fibronectin expression (red, A, D) is expressed similarly in forelimb (A–C) and hindlimb (D–F) day 3 micromasses. Cultures are counterstained with DAPI for nuclei (blue, B, E), scale = 70μM. G; Tenascin—C protein levels are equivalent in mouse 11.5dpc forelimb and hindlimb buds by western blot. Equal protein loading is shown by reprobing for α–tubulin. (TIF) [file pone.0180453.s001.tif]
